# Multisubject “Learning” for Mental Workload Classification Using Concurrent EEG, fNIRS, and Physiological Measures

**DOI:** 10.3389/fnhum.2017.00389

**Published:** 2017-07-27

**Authors:** Yichuan Liu, Hasan Ayaz, Patricia A. Shewokis

**Affiliations:** ^1^School of Biomedical Engineering, Science and Health Systems, Drexel University Philadelphia, PA, United States; ^2^Cognitive Neuroengineering and Quantitative Experimental Research Collaborative, Drexel University Philadelphia, PA, United States; ^3^Department of Family and Community Health, University of Pennsylvania Philadelphia, PA, United States; ^4^Division of General Pediatrics, Children's Hospital of Philadelphia Philadelphia, PA, United States; ^5^Nutrition Sciences Department, College of Nursing and Health Professions, Drexel University Philadelphia, PA, United States

**Keywords:** fNIRS, EEG, heart rate variability, respiration rate, n-back, mental workload, multimodal fusion, brain computer interface

## Abstract

An accurate measure of mental workload level has diverse neuroergonomic applications ranging from brain computer interfacing to improving the efficiency of human operators. In this study, we integrated electroencephalogram (EEG), functional near-infrared spectroscopy (fNIRS), and physiological measures for the classification of three workload levels in an n-back working memory task. A significantly better than chance level classification was achieved by EEG-alone, fNIRS-alone, physiological alone, and EEG+fNIRS based approaches. The results confirmed our previous finding that integrating EEG and fNIRS significantly improved workload classification compared to using EEG-alone or fNIRS-alone. The inclusion of physiological measures, however, does not significantly improves EEG-based or fNIRS-based workload classification. A major limitation of currently available mental workload assessment approaches is the requirement to record lengthy calibration data from the target subject to train workload classifiers. We show that by learning from the data of other subjects, workload classification accuracy can be improved especially when the amount of data from the target subject is small.

## Introduction

Mental workload refers to the cognitive and psychological effort required to complete given tasks. Continuous evaluation of mental workload enables real-time adjustment in the task load assigned to human operators so that their workload can be kept at a moderate level for improving human performance (Parasuraman et al., 1992; Parasuraman, 2003). Studies have thus far mainly decoded human workload levels from brain activity electroencephalogram (EEG) measures (Gevins et al., 1998; Brouwer et al., 2012). Cerebral hemodynamics have recently gained attention for applications in brain-computer interfaces (Naseer and Hong, 2015) and the decoding of mental workload level with the emergence of the portable measurement technique known as functional near-infrared spectroscopy (fNIRS) (Sassaroli et al., 2008; Ayaz et al., 2012; Herff et al., 2014). Previous studies have adopted a combination of EEG and non-brain measures such as heart rate variability, respiration rate, and eye movement (Hankins and Wilson, 1998; Wilson and Russell, 2003; Fairclough, 2009) for mental workload assessment. Moreover, results from our previous study suggests that when combining EEG and fNIRS workload classification accuracies, they outperform the EEG-alone and fNIRS-alone results in mental workload level classification (Liu et al., 2017).

Before mental workload can be decoded from brain and body signals, it is typical that a time-consuming calibration process is required to derive a decoder for each individual operator. This is primarily due to the challenge that psychophysiological signals vary considerably between different people and over time. In the traditional calibration approach, lengthy psychophysiological signals (i.e., calibration data) need to be recorded from an operator so that a decoder can learn both the signal patterns specific to this operator and the variations of these patterns over time.

This problem is not unique to mental workload decoding. The lengthy calibration process is also required to decode other types of mental activities such as motor imagery (Blankertz et al., 2006). To address this problem for motor imagery decoding, Lotte and Guan proposed an alternative calibration approach (Lotte and Guan, 2010). In this approach, a decoder is derived using calibration data from both the target subject and some other subjects. Lotte and Guan argued that despite the large inter-subject variations, similar signal patterns can be found across some individuals so that less calibration data from the target subject is required to derive a decoder. This approach has been further investigated by other researchers, with positive results (Devlaminck et al., 2011; Samek et al., 2013). An alternative approach to learning from other subjects, is to identify which models incorporate the inter-subject variations from a large database (Fazli et al., 2009).

For mental workload decoding, only one preliminary study to date has explored the reduction of calibration time using a simulated aviation task (Wang et al., 2012). Authors have shown that calibrating decoders using data from both the target subject and a pool of other subjects did not degrade the decoding accuracies compared to using data only from the target subject. However, no benefit of including data from the other subjects has been shown.

In this study, the integration of EEG, fNIRS, and physiological signals was investigated for the classification of three workload levels induced by the n-back working memory task. The objective was two-fold: first, to compare the classification performance using the different modalities and their combinations; and, second, to investigate learning in a workload decoder using data from other subjects as an approach to improve workload classification performance when the sample size of the target subject is small.

## Materials and methods

### Participants

A total of 25 volunteers were recruited for participation in this study. Two of the participants were unable to finish the protocol. Another two participants were rejected from the analyses due to excessive movement. Consequently, a total of 21 valid subjects [all right-handed, 12 female, ages 25.9 ± 4.9 (mean ± *SD*)] were included in the analysis. The Edinburgh Handedness Inventory (Oldfield, 1971) showed that participants were right handed and the average Laterality Quotient (L.Q.) and Decile is 78.7 ± 22.2 and 6.2 ± 3.4, respectively. Participants self-reported that they had their vision corrected to 20/20, did not have any history of neurological or psychiatric disorders and that they did not take any medication known to affect brain activity. Prior to the experiment, participants gave written informed consent for their participation in the study. The protocol was approved by the Institutional Review Board of Drexel University.

### Recording

**Figure 1:**
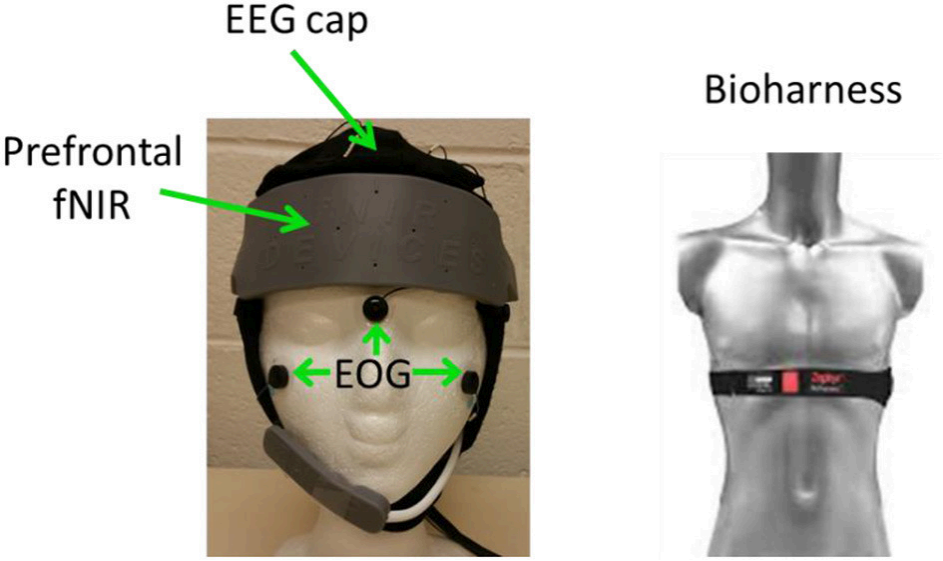
Recording setup.

EEG, fNIRS, Heart rate, R-R interval, breath rate, and breath depth were simultaneously recorded during data collection. Figure 1 shows an overview of the recording setup.

**Figure 2:**
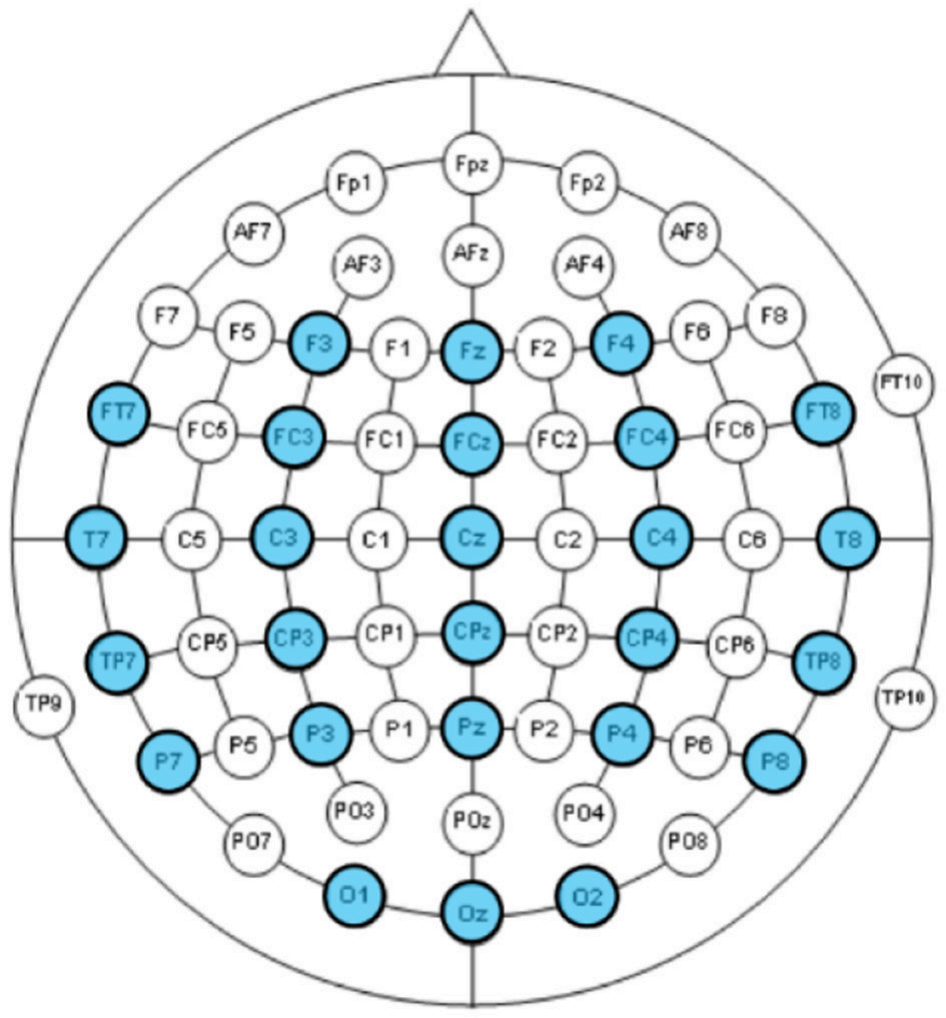
EEG channels according to the International 10–10 system. The 26 recorded channels were highlighted.

**EEG** were recorded using a Neuroscan Nuamp amplifier by Compumedics Neuroscan (http://compumedicsneuroscan.com/) from 26 locations according to the International 10–10 system (See Figure 2). Three additional electrodes, one placed above Nasion, the other two placed below the left/right outer canthus were used for electrooculography (EOG) artifact correction according to Schlögl et al. (2007). All 29 channels (26 EEG + 3 EOG) were band-pass filtered 0.1–100 Hz, digitally sampled at 500 Hz and referenced to a linked mastoid.

**Figure 3:**
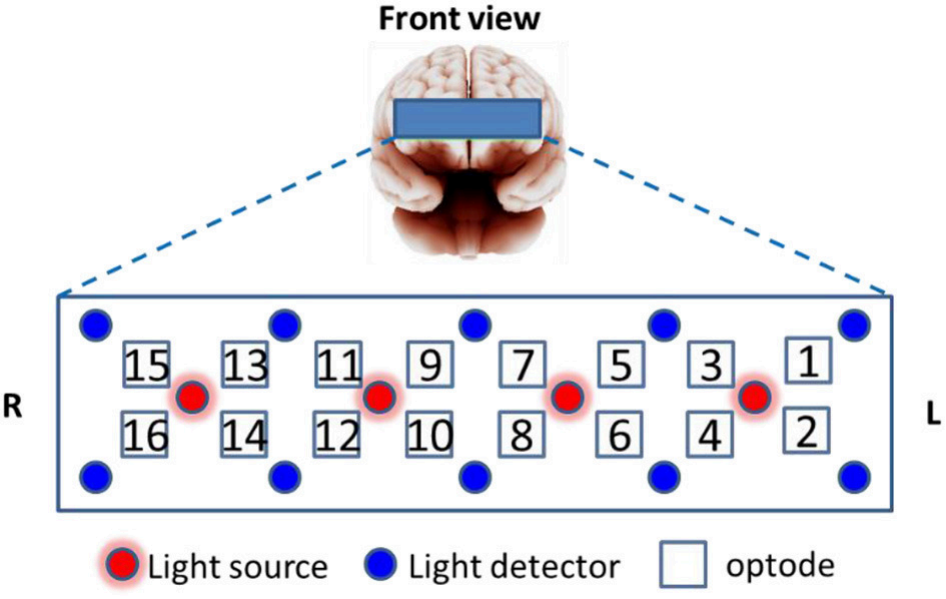
fNIRS sensor layout with 16 optodes from prefrontal cortex.

**Prefrontal fNIRS** were recorded from the forehead at a 2 Hz sampling rate using a 16-optode continuous wave fNIRS system developed at Drexel University (Ayaz et al., 2012, 2013) and manufactured by fNIR Devices LLC (http://fnirdevices.com/). The sensor included 4 light sources (LED) that can emit 730 and 850 nm wavelength light and 10 photon detectors (See Figure 3). The distance between light sources and detectors was 2.5 cm which allowed for a ~1.2 cm penetration depth. To ensure repeatable sensor placement, the center of the sensor was aligned to the midline and the bottom of the sensor touched the participant's eye brow.

**Systemic NIR** were recorded from the right cheek at a 4 Hz sampling rate using a 2-optode continuous wave wireless fNIRS system developed at Drexel University (Ayaz et al., 2013) and manufactured by fNIR Devices LLC. The systemic NIR was not used in the current study.

**Heart rate, R-R interval, breath rate, and breath depth** were recorded using a Zephyr Bioharness chest band (https://www.zephyranywhere.com/).

### Experiment

Subjects sat comfortably in front of an LED screen. Sequences of capitalized letter stimuli (~1.7° visual angle) were shown on the center of the screen. The BCI2000 software was employed for stimulus delivery and for the recording of EEG and behavioral data (Schalk et al., 2004). Each letter was displayed for a duration of 480 ms and the inter-stimulus interval (ISI) was 2,520 ms. Subjects were instructed to click a keypad button with their right index finger in response to a “match stimulus” and click another keypad button with their right middle finger in response to a “non-match stimulus” as fast as possible. There were three workload conditions. In the 0-back condition, letter “X” was the match. In the 2-back condition, a letter was the match if it was shown two screens back. In the 3-back condition, a letter was the match if it was shown three screens back.

**Figure 4:**
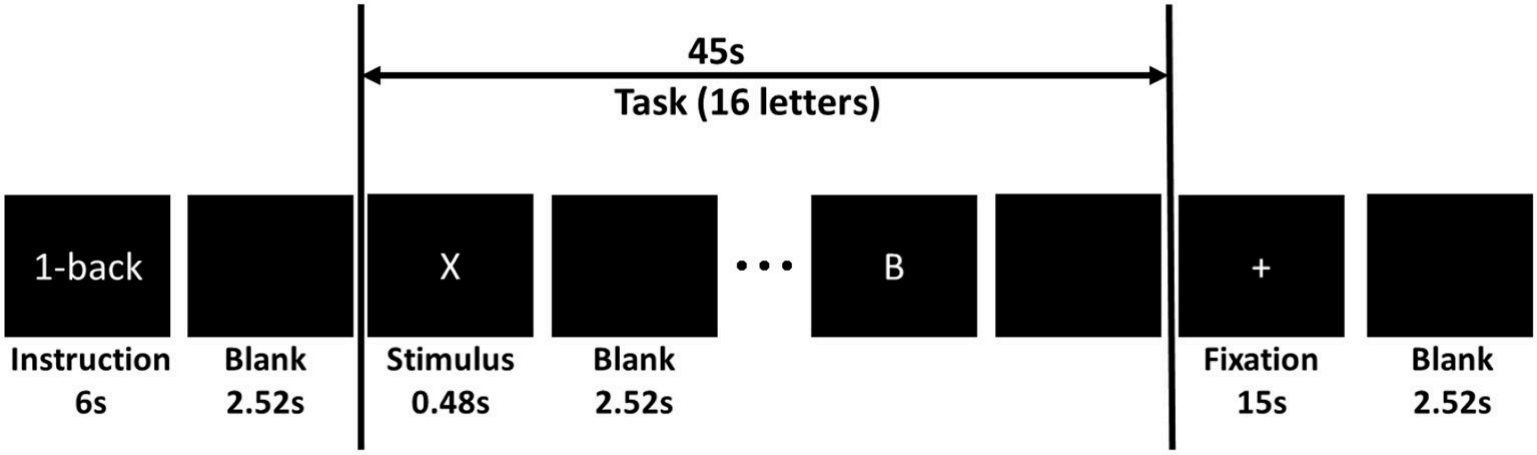
Timeline of an n-back block.

The letter stimuli were grouped into n-back blocks. Each block included 6 s of instruction, 45 s of task performance, and 15 s of fixation. The instruction period informed the subject which task (0-, 2-, or 3-back) to perform. During the task period, 15 letters were shown to the participants on the screen in a pseudo random order. Four of the letters were targets. No letters appeared more than twice in succession within a block. In the fixation period, subjects were instructed to focus their eye gaze on a white plus sign located at the center of the screen allowing fNIRS signals to return to the baseline. Figure 4 shows the time line of a typical n-back block.

**Figure 5:**
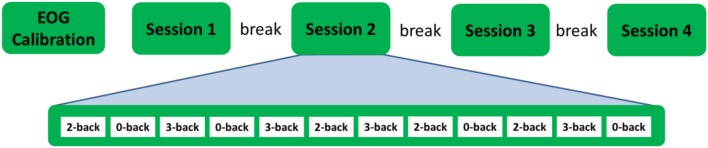
Experiment outline.

There were four recording sessions. Each session included 12 n-back blocks, 4 from each condition. Hence, there were 48 n-back blocks for the entire experiment, 16 from each condition. To reduce the correlation between adjacent samples and to balance time induced experimental factors such as fatigue across the three workload conditions, the 48 n-back blocks were grouped into 16 repetitions. Each repetition included one block from each workload conditions. The order of the blocks was further randomly shuffled so that no workload condition was repeated twice in succession within a session. Before the start of the first session, subjects practiced one block from each condition for familiarization with the procedure and an ~5 min long EOG calibration session was performed during which subjects were instructed to rotate, blink and move (up/down, left/right) their eyes. A 5 min break was given to the subjects between the recording sessions. The entire recording time was about 1 h. Figure 5 shows the outline of the experiment.

### EEG signal processing

In this work, we extracted for each EEG channel the band powers of 1–3, 4–7, 8–12, 13–19, and 20–30 Hz bandwidths. This was performed at a single stimulus level, forming a feature vector *f*_*EEG*_ of *6 bands* × *26 channels* = *156* length for each of *the 48 blocks* × *15 stimuli* = *720* sample epochs for each subject.

Raw EEG and EOG signals are band-pass filtered 1–35Hz. A regression-based approach was adopted to reduce EOG contamination by using the calibration data recorded before the n-back sessions started (Schlögl et al., 2007). Epochs were extracted 0–2.8 s and baseline corrected −0.2 to 0 s with respect to stimulus onset. The power spectral density of each epoch was then estimated using the Multitaper method (Thomson, 1982) with 8 Discrete Prolate Spheroidal Sequences (DPSS) window of 3 s long for subsequent analysis.

### fNIRS signal processing

The average oxygenated hemoglobin (oxy-Hb) and deoxygenated hemoglobin (deoxy-Hb) amplitude change between (25 s, 45 s) and (−5 s, 5 s) with respect to the block start was used as a feature. The features were extracted from 14 forehead optodes, forming a feature vector *f*_*NIR*_ of *14* × *2(oxy/deoxy-Hb)* = *28* length for each of the *48 sample blocks*. Optode 1 and 15 were rejected from analysis because they are over the hairline for most of the subjects. The average activation amplitude with respect to a baseline was adopted as the feature for characterizing the mental activities in many studies (Ayaz et al., 2007, 2012; Merzagora et al., 2009; Herff et al., 2012; Liu et al., 2013). This feature extraction strategy has been shown to be more reliable when compared to other possible feature choices in our preliminary analysis.

Raw light intensities recorded from prefrontal fNIRS were first visually inspected to reject those optodes with inadequate contact or those positioned over the hairline. Raw light intensities were converted into concentration changes in oxy-hemoglobin (oxy-Hb) and deoxy-hemoglobin (deoxy-Hb) using the modified Beer-Lambert law (Cope and Delpy, 1988). Oxy-Hb/deoxy-Hb signals are band-pass filtered at 0.005–0.1 Hz for reducing artifacts from physiological signals (e.g., heartbeat and respiration) before subsequent analysis.

### Heart rate variability (HRV) processing

Heart rate variability (HRV) was estimated according to Clifford (2002) and Gritti et al. (2013). The R-to-R interval recorded by the Bioharness was first interpolated to form a 4 Hz time series. Epochs were extracted for each n-back block with (0 s, 45 s) time windows with respect to the onset of the first stimulus and the power spectral density (PSD) were estimated using a single DPSS window of 45 s long (Thomson, 1982) for evaluating the variability of the R-to-R interval. The average PSD in the bandwidths 0.02–0.06 Hz (mainly originated from body temperature regulation), 0.07–0.14 Hz (related to regulation of blood pressure), and 0.15–0.5 Hz (momentary respiratory influences on heart rate) were extracted as suggested by Scerbo et al. (2001).

In addition to HRV, the average of heart rate, breath rate, and breath depth for each n-back block recorded by Bioharness were extracted as features.

### Multimodality workload classification

**Figure 6:**
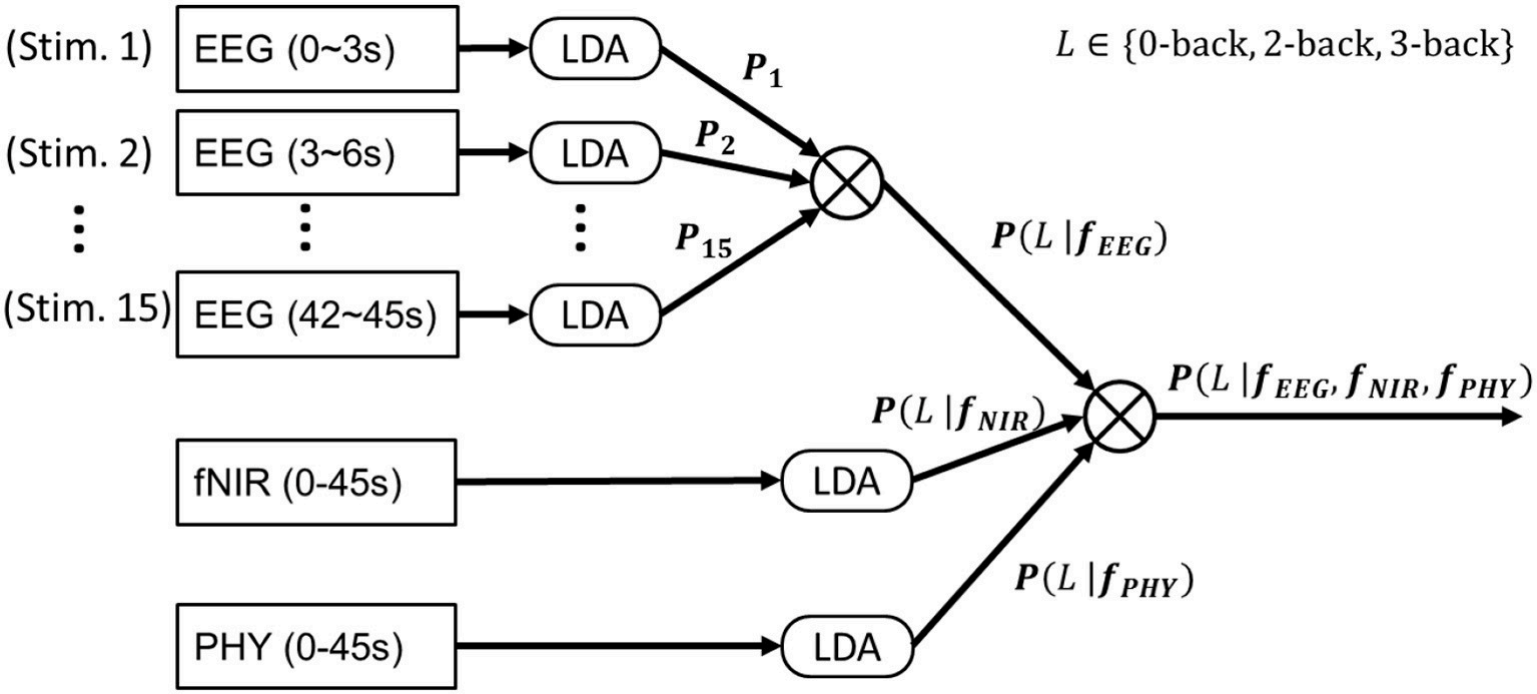
EEG+fNIRS+PHY workload classification. A Linear Discriminant Analysis (LDA) was trained to classify EEG band power features at the single stimulus level (4 s epoch). The output probabilities from the 15 stimuli (of a block) were Naïve-Bayes combined to produce *P*(*L*|*f*_*EEG*_). A second LDA was trained to classify fNIRS features extracted from each block (45 s epoch) to produce *P*(*L*|*f*_*NIR*_). A third LDA was trained to classify PHY features extracted from each block to produce *P*(*L*|*f*_*PHY*_). *P*(*L*|*f*_*EEG*_), *P*(*L*|*f*_*NIR*_), and *P*(*L*|*f*_*PHY*_) were Naïve-Bayes combined for EEG+fNIRS+PHY classification. All of the above procedures were conducted on calibration data. The LDA classifiers were then applied on testing data to evaluate the classification performance.

We considered the three-class classification problem of 3- vs. 2- vs. 0-back. A multiclass linear discriminant analysis (LDA) was adopted for classification. To prevent a covariance matrix from becoming singular due to small sample size, an automatic shrinkage using the Ledoit-Wolf lemma (Schafer and Strimmer, 2005) was adopted. The following eight different classifications were considered dependent on the adopted modalities (See Figure 6):

1. **EEG-alone**. A LDA was trained to classify EEG features at the single stimulus level (3 s time window with respect to a single stimulus). At the block level (45 s time window, included 15 stimuli), the LDA predicted probabilities for each of the 15 stimuli were Naïve-Bayes combined (Kuncheva et al., 2001) to produce *P*(*L*|*f*_*EEG*_) where *L* ∈ {0*-back*, 2*-back*, 3*-back*}, which determined the predicted workload levels. More specifically, in Naïve-Bayes fusion, the product of the predicted probabilities from the 15 stimuli was calculated and normalized as follows:
  (1) $$P\left(L|f_{EEG}\right)=\frac{\prod_{i\;=\;1}^{15}P\left(L|f_{EEG}^{i}\right)}{\prod_{i\;=\;1}^{15}P\left(0\text{​-​}back|f_{EEG}^{i}\right)+\prod_{i\;=\;1}^{15}P\left(2\text{​-​}back|f_{EEG}^{i}\right)+\prod_{i\;=\;1}^{15}P\left(3\text{​-​}back|f_{EEG}^{i}\right)}$$
2. **fNIRS-alone**. A LDA was trained to classify fNIRS features at the block level. The LDA probability output was termed *P*(*L*|*f*_*NIR*_).
3. **PHY-alone**. A LDA was trained to classify PHY features (HRV, heart rate, respiration rate, and respiration depth) at the block level. The LDA probability output was termed *P*(*L*|*f*_*PHY*_).
4. **EEG**+**fNIRS**. *P*(*L*|*f*_*EEG*_) and *P*(*L*|*f*_*NIR*_) were Naïve-Bayes combined for a final decision. More specifically, the product of the predicted probabilities from the two modalities was calculated and the output class *c* was determined as follows:
  (2) $$c=arg\;max_{L}[P\left(L\text{|}f_{EEG}\right)\cdot P\left(L\text{|}f_{NIR}\right)]$$
5. **EEG**+**PHY**. *P*(*L*|*f*_*EEG*_) and *P*(*L*|*f*_*PHY*_) were Naïve-Bayes combined for a final decision.
6. **fNIRS**+**PHY**. *P*(*L*|*f*_*NIR*_) and *P*(*L*|*f*_*PHY*_) were Naïve-Bayes combined for a final decision.
7. **EEG**+**fNIRS**+**PHY**. *P*(*L*|*f*_*EEG*_), *P*(*L*|*f*_*NIR*_) and *P*(*L*|*f*_*PHY*_) were Naïve-Bayes combined.

### Learning from other participants

We consider the following calibration approaches:

- **Traditional calibration**, which derives a classifier only with data from a specific subject. In terms of a LDA classifier, the mean and covariance matrix of the feature vector μ_*i*_ and Σ were estimated from a feature matrix extracted from the data of a target subject for estimating the posterior probability of a class given a feature vector. To obtain good classification accuracy, μ_*i*_ and Σ need to be estimated from a large data set recorded during a lengthy calibration session.
- **Proposed calibration**, which derives a classifier with data from both a specific subject and a pool of other subjects. As Lotte and Guan proposed, μ_*i*_ and Σ can be estimated from the target subject and a pool of other subjects to reduce the calibration time of the target subject (Lotte and Guan, 2010). For each subject, the features were first z-score transformed to reduce the between-subject variations. For the target subject, only the training data was used for estimating the mean and variance of each feature. The mean and covariance matrix of the feature vector of each subject was then estimated. Finally, the mean and covariance matrices from all subjects were combined according to Equation (3) and Equation (4).

(3) $$\mu_{i}=\left(1-\lambda \right)\mu_{i}^{t}+\lambda \frac{1}{\left|S_{t}\left(\Omega \right)\right|}\sum_{j\in \;S_{t}\left(\Omega \right)}\mu_{i}^{j}\;$$

(4) $$\Sigma =\left(1-\lambda \right)\Sigma^{\text{t}}+\lambda \frac{1}{\left|S_{t}(\Omega )\right|}\sum_{j\in \;S_{t}(\Omega )}\Sigma^{\text{j}}\;$$

where $\mu_{i}^{t}$ and Σ^t^ are the mean and covariance estimated from the target subject, *S*_*t*_(Ω) is a set of subjects that does not include the target subject (leave-one-subject-out) and λ ∈ [0, 1] was a parameter representing the weight of other subjects. In this study, λ was empirically chosen to be 0.5.

When the sample size from the target subject is small, we expect that an improved classification performance can be achieved by incorporating the mean and covariance matrices estimated from other subjects.

### Performance evaluation

A repeated learning-testing method (Burman, 1989) was adopted for performance evaluation. The procedure was done as follows:

For subject *j* = 1, …, 21:

1. For *iteration i* = 1, …, 100:
  - Data splitting:
    The data of the target subject *j* were randomly split into a calibration set and a testing set three times with varying calibration sample size:
    - 13 min calibration (12 samples), 39 min testing (36 samples).
    - 26 min calibration (24 samples), 26 min testing (24 samples).
    - 39 min calibration (36 samples), 13 min testing (12 samples).
  - Classifier calibration:
    - For traditional calibrations, the calibration sets were used to train the classifiers using LDA.
    - For the proposed calibrations, the calibration sets and data from all other subjects were used to train the classifiers.
  - Classifier evaluation:
    - The testing sets were used to evaluate the classification accuracy.
2. For each of the evaluated approaches, testing accuracies from the 100 iterations are averaged for a stable performance evaluation.

### Multiple comparisons

To correct for multiple testing, we adopted false discovery rate (FDR) control with the Benjamini-Hochberg procedure (Benjamini and Hochberg, 1995). Without specification, we rejected null hypotheses for FDR *q* < 0.05.

## Results

### Behavior performance

To verify the successful manipulation of workload level with the adopted protocol, we evaluated the following three behavior measures:

1. **d-prime**, which was the key-press true positive rate minus false positive rate:
  (5) $$\begin{array}{l} d\text{-​​}prime=\;\frac{N(stim=match\;and\;responded=match)}{N(stim=match)}\\ \;-\frac{N(stim=non-match\;and\;responded=match)}{N(responded=match)} \end{array}$$
  where *N*(*event*) is the number of cases of an event, *stim* = *match*/*unmatch* represent the true stimulus type, and *responded* = *match*/*unmatch* represent subject's response.
2. **Accuracy**, which was the key-press accuracy.
3. **Response delay**: The time elapsed between stimulus onset and key-press.

**Figure 7:**
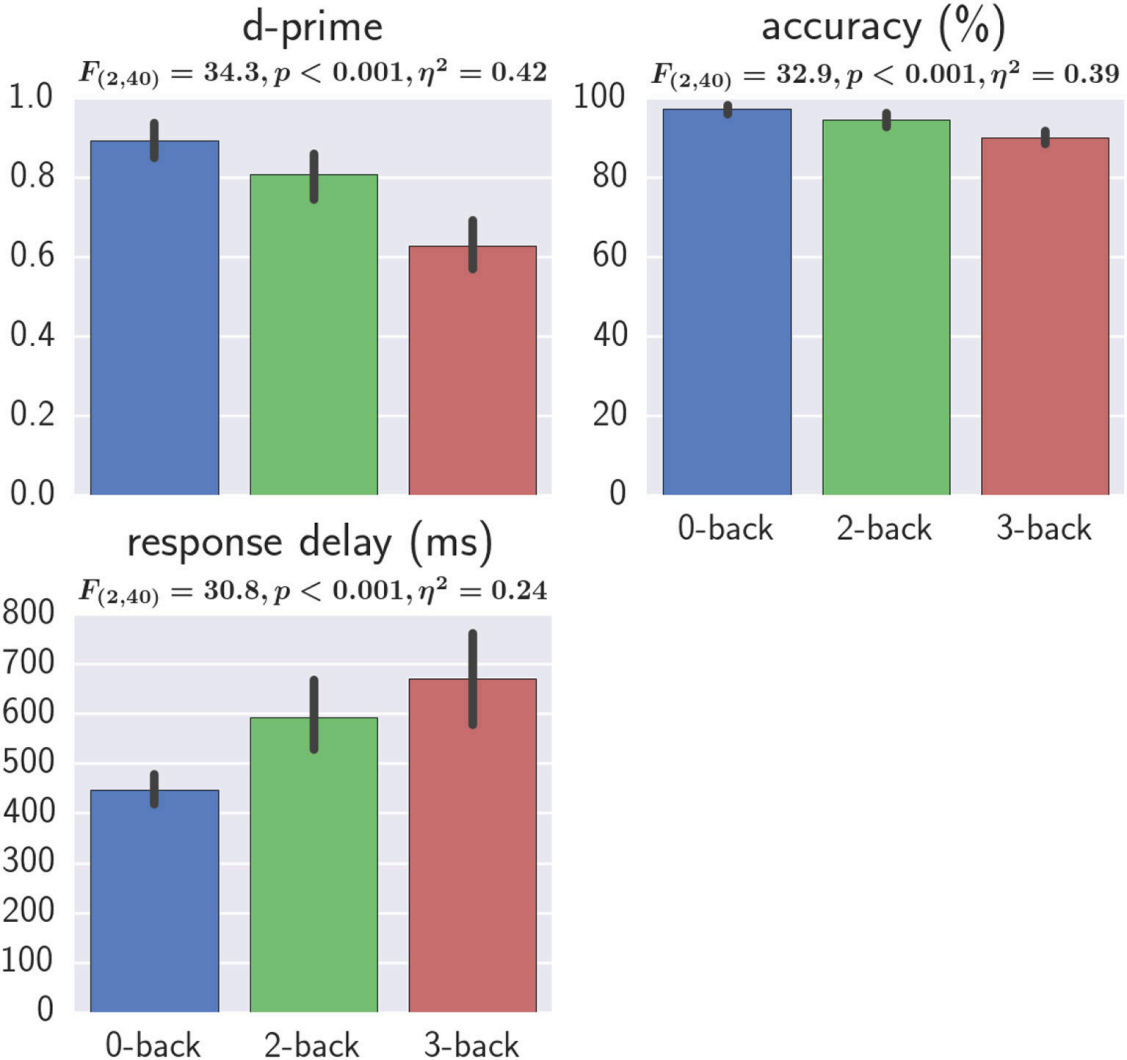
Effect of workload on behavioral results. One-way repeated measures ANOVA results and the η^2^ effect sizes with workload as the independent variable are shown. Error bars represent the bootstrapped 95% confidence interval.

For all three behavioral measures, one-way repeated measures ANOVAs revealed a significant effect of workload and *post-hoc* tests revealed significant differences (FDR *q* < 0.05) between all three workload levels, suggesting the successfully manipulation of workload level (Figure 7). The generalized eta-squared (η^2^) as reported by the ezANOVA library of R was used (Bakeman, 2005).

### Effect of workload on EEG band powers

**Figure 8:**
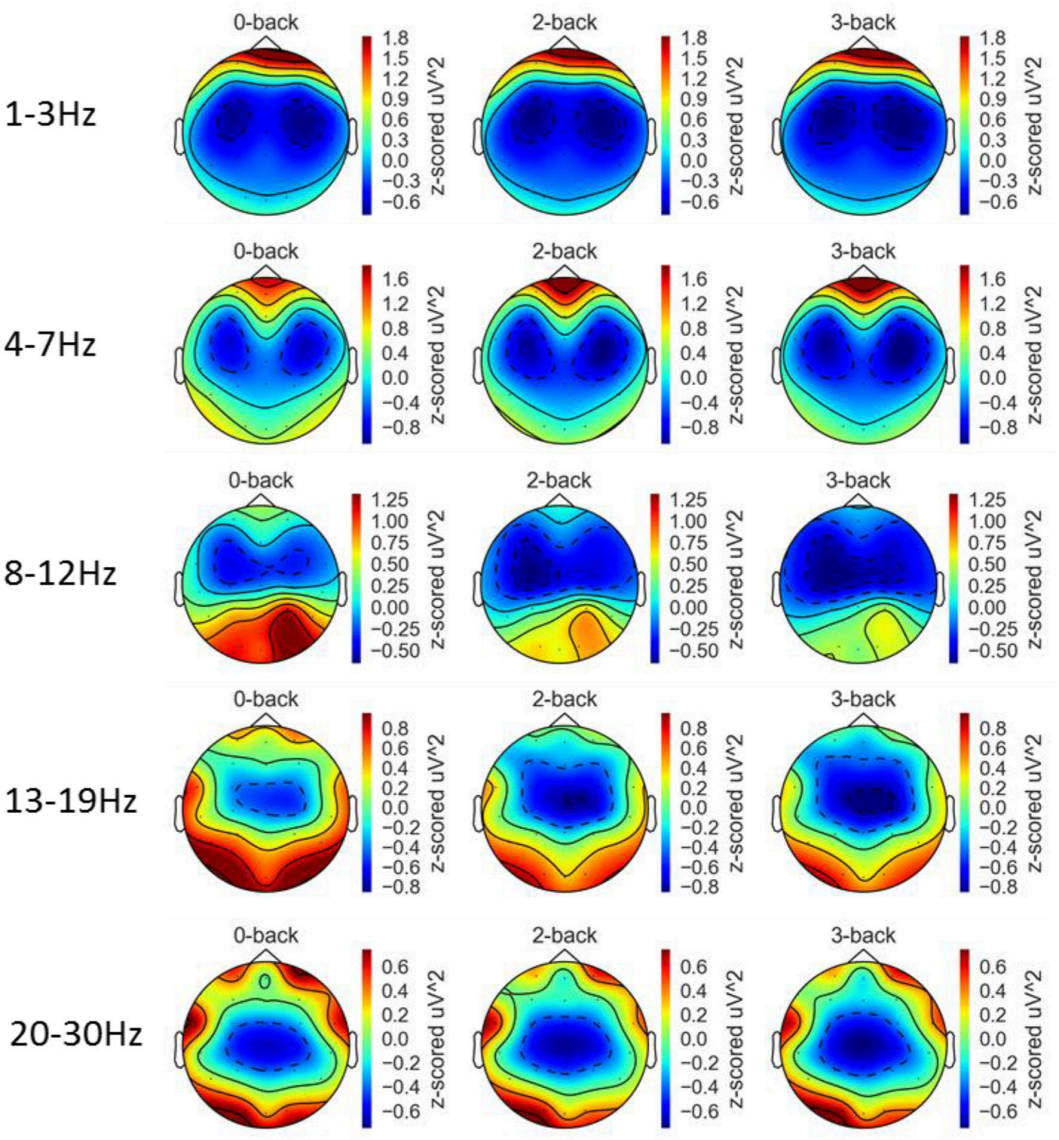
Topographic maps of EEG band powers at the five frequency bands and three workload conditions averaged over 21 participants.

Figure 8 depicts the topographic map of EEG band powers. A repeated measures ANOVA was applied to assess the effect of workload on the six mid-line channels Fz, FCz, Cz, CPz, Pz, and Oz and the results are shown in Table 1. For delta activity, a significant effect of workload was found at Cz and CPz (FDR *q* < 0.05) where the delta band power decreased with increased workload. Workload had a significant effect on theta band at channel Fz and Cz (FDR *q* < 0.05). At Fz, theta band increased with increased workload whereas at Cz, theta band power decreased with increased workload. Workload had a significant effect on alpha band power at all of the six midline channels Fz, FCz, Cz, CPz, Pz, and Oz (FDR *q* < 0.05). At all six channels, alpha band power decreased with increased workload. Workload has a significant effect on low beta band power at the six midline channels Fz, FCz, Cz, CPz, Pz, and Oz (FDR *q* < 0.05). At all six channels, low beta band power decreased with increased workload. Workload had a significant effect on high beta band power at Fz, FCz, Cz, CPz, and Pz (FDR *q* < 0.05). At these five channels, high beta band power decreased with increased workload. The significant effects that has been found in the low and high beta band may be confounded by motor responses as the 13–30 Hz range is typically associated with motor responses (Pfurtscheller et al., 1996, 2006). To investigate the effect of motor responses, a 2 (key press type: middle/index finger) × 3 workload level (0-/2-/3-back) ANOVA with repeated measures on both factors was conducted using the amount of key-press responses as the dependent. No significant effect of workload level [F_(2, 42)_ = 0.83, *p* = 0.44, η^2^ < 0.01] or the interaction between key-press type and workload level [F_(2, 42)_ = 2.01, *p* = 0.15, η^2^ = 0.03] was found. Mean and standard deviations of the number of key-presses within each block across the 21 participants for each of the three workload conditions can be found in Supplementary Table 1.

**Table 1:** Effect of workload on EEG band powers.

	**Delta**	**Theta**	**Alpha**	**Low beta**	**High beta**
	**F_(2, 40),_ η^2^**	**F_(2, 40),_ η^2^**	**F_(2, 40),_ η^2^**	**F_(2, 40),_ η^2^**	**F_(2, 40),_ η^2^**
Fz	0.3, 0.01	**3.5**, **0.15**	**21.2**, **0.51**	**67.3**, **0.77**	**17.8**, **0.47**
FCz	2.9, 0.13	2.1, 0.10	**23.5**, **0.54**	**66.4**, **0.77**	**22.6**, **0.53**
Cz	**7.3**, **0.27**	**5.9**, **0.23**	**14.5**, **0.42**	**98.9**, **0.83**	**31.6**, **0.61**
CPz	**3.9**, **0.16**	1.7, 0.08	**13.9**, **0.41**	**85.8**, **0.81**	**31.9**, **0.61**
Pz	1.0, 0.05	1.5, 0.07	**13.4**, **0.40**	**73.0**, **0.78**	**28.6**, **0.59**
Oz	0.1, <0.01	2.7, 0.12	**14.0**, **0.41**	**10.7**, **0.35**	1.0, 0.05

*F-ratio statistics and the η^2^ effect sizes are reported. Bold highlighted results are significant after correcting for multiple comparisons using FDR (Benjamini and Hochberg, 1995) (FDR q < 0.05)*.

*Interpretation of the effect size η^2^ are 0.02 = small, 0.13 = medium, and 0.26 or greater is large (Bakeman, 2005)*.

### Effect of workload on fNIRS measures

**Figure 9:**
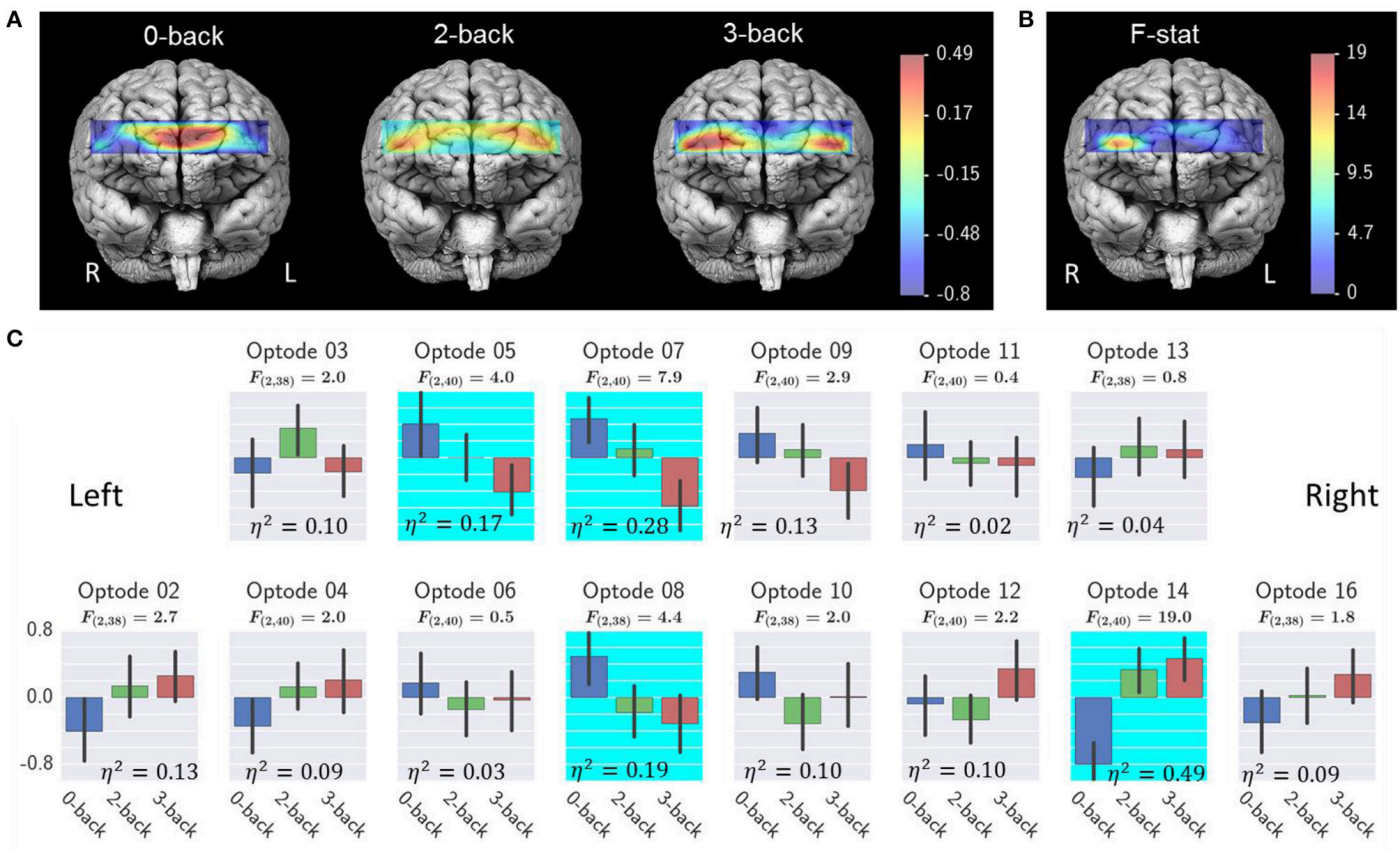
Effect of workload on fNIRS oxy-Hb. **(A)** Average fNIRS oxy-Hb at each workload level; **(B)** ANOVA F- ratio statistics and η^2^ effect size of workload effect; **(C)** Average oxy-Hb for each optode. Highlighted optodes showed a significant workload effect (optode 5, 7, 8, and 14, FDR *q* < 0.05). Error bars illustrated the bootstrapped 95% confidence interval. Interpretation of the effect size η^2^ are 0.02 = small, 0.13 = medium, and 0.26 or greater is large (Bakeman, 2005).

Figure 9 shows the results from oxy-Hb. A common average reference approach was applied to remove the average oxy-Hb across all optodes and from each individual optode for reducing the effect of systemic physiological artifacts. Repeated measures ANOVA revealed a significant effect of workload on optode 5, 7, 8, and 14. *Post*-*hoc* tests revealed a significant 3-back > 0-back and 2-back > 0-back at optode 14. A optode 7, there was a significant effect of 3-back < 0-back and 3-back < 2-back. At optode 8, there was a significant effect of 3-back < 0-back and 2-back < 0-back. No significant *post-hoc* test results were detected at optode 5.

### Effect of workload on physiological measures

**Figure 10:**
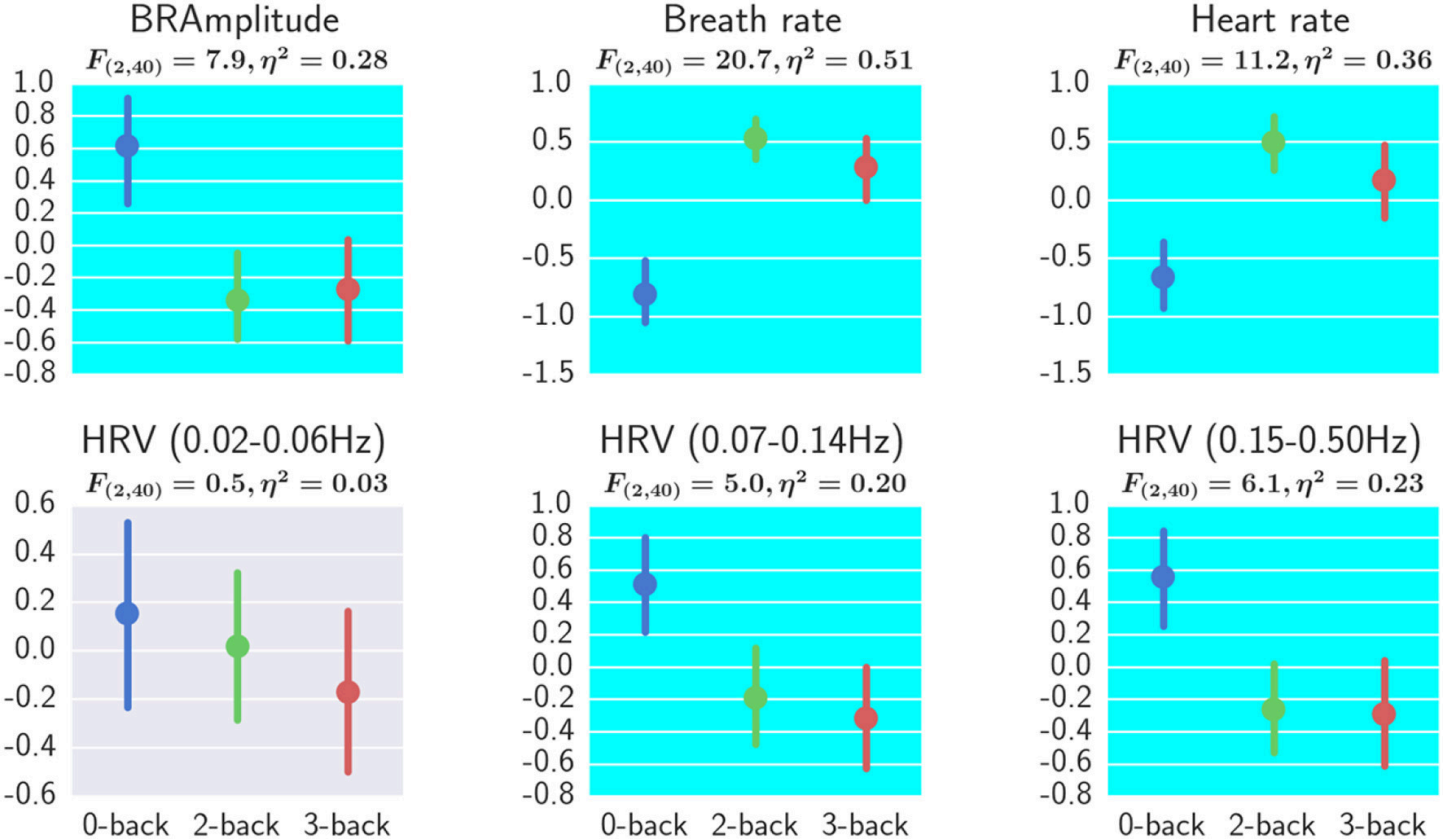
Effect of workload on physiological measurements. One-way repeated measures ANOVA results and the η^2^ effect sizes with workload as the independent variable are shown. All measures except for HRV (0.02–0.06 Hz) showed a significant effect of workload (FDR *q* < 0.05). Error bars represented the bootstrapped 95% confidence interval. BRAmplitude, breath amplitude. Interpretation of the effect size η^2^ are 0.02 = small, 0.13 = medium, and 0.26 or greater is large (Bakeman, 2005).

The effect of workload on physiological measurements are shown in Figure 10. For each subject, the average heart rate at 0-back, 2-back, and 3-back blocks were calculated respectively and the three heart rate values were z-score standardized before analysis. The same preprocessing procedure was applied to the other physiological measurements: breath rate, breath amplitude and HRV. A repeated measures ANOVA revealed a significant effect of workload on breath amplitude, breath rate, heart rate, HRV 0.07–0.14 Hz, and HRV 0.15–0.5 Hz (FDR *q* < 0.05). *Post-hoc* tests revealed significant differences between 3-back and 0-back and also between 2-back and 0-back for breath amplitude, breath rate, heart rate HRV 0.07–0.14 Hz, and HRV 0.15–0.5 Hz (FDR *q* < 0.05).

### Workload classification

**Figure 11:**
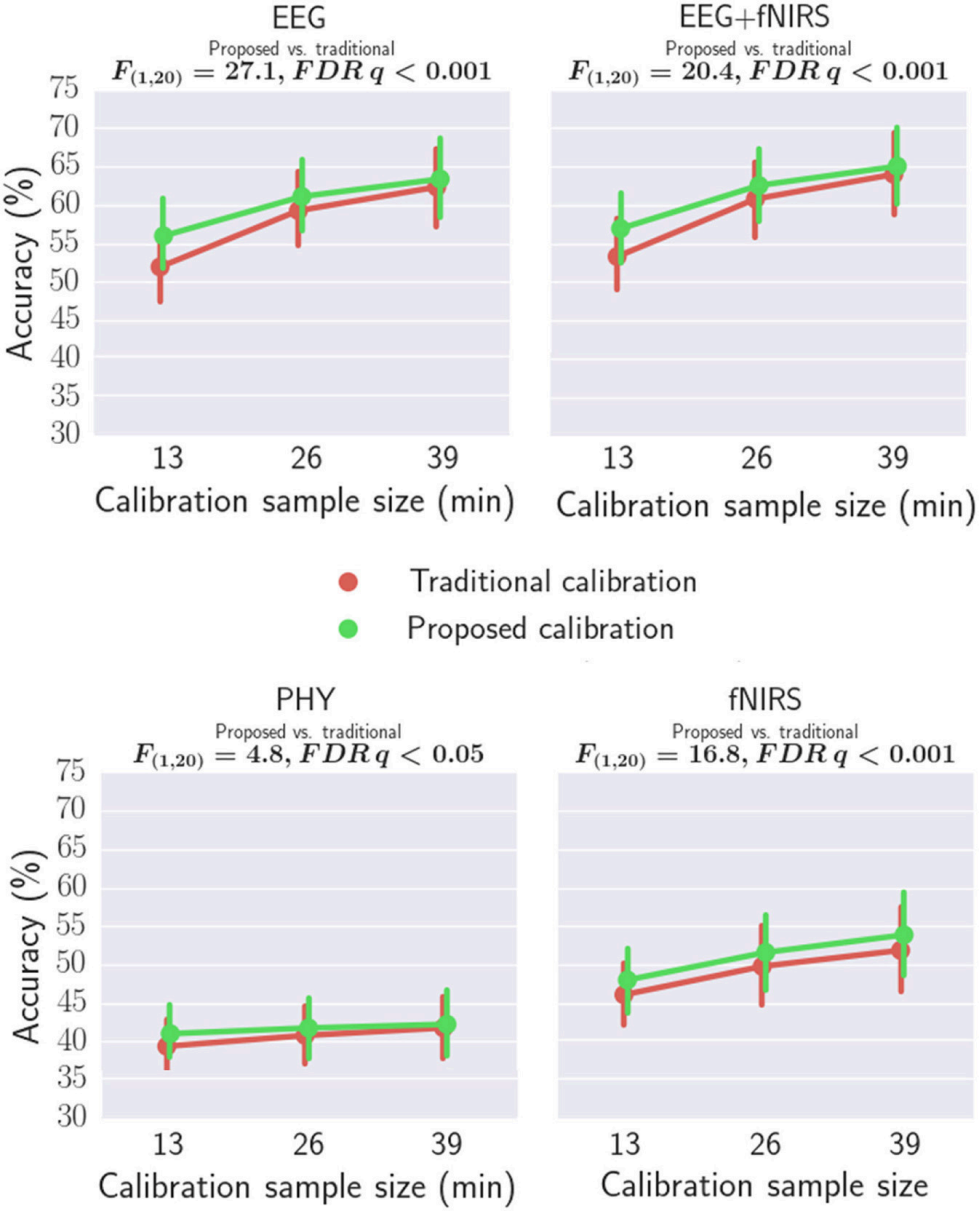
Comparing the classification accuracies of traditional and proposed calibration approaches using EEG, fNIRS, PHY, and EEG+fNIRS.

**Figure 12:**
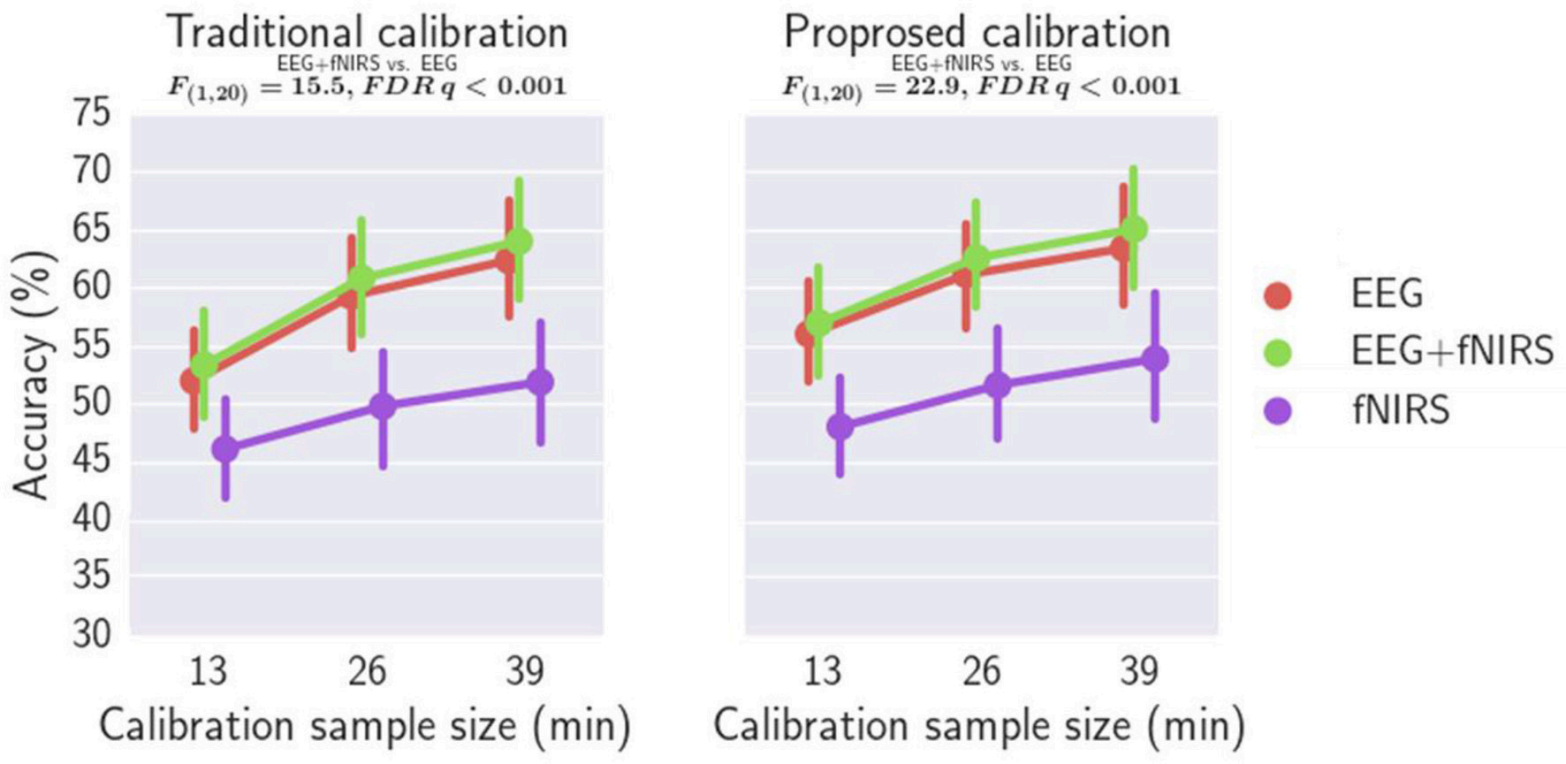
Comparing the classification accuracies of EEG+fNIRS multimodal model and EEG-alone model.

Workload classification results are shown in Table 2, Figures 11, 12 and Supplementary Tables 2, 3. For all investigated approaches and with the different calibration sample sizes, classification accuracy was significantly better than chance level (33.3%) as revealed by one-tailed Wilcoxon signed rank tests. Figure 11 compares the accuracy using traditional and proposed calibration approaches. The results of the repeated measures ANOVAs indicate that the proposed calibration approach significantly outperforms the traditional calibration approach for EEG-based classification, fNIRS-based classification, physiological based classification, and EEG-fNIRS multimodal classification (*p* < 0.05). The effect size of the results are shown in Table 3. *Post-hoc* analysis was conducted using a Wilcoxon Signed Rank test with FDR correction and the results are shown in Supplementary Table 4. For the calibration sample size of 13 min, the proposed calibration approach significantly outperformed the traditional calibration approach for EEG-alone, fNIRS-alone, PHY-alone, and EEG+fNIRS (FDR *q* < 0.05). For the calibration sample size of 26 min, the proposed calibration approach significantly outperformed the traditional calibration approach for EEG-alone, fNIRS-alone, and EEG+fNIRS (FDR *q* < 0.05). While for the calibration sample size of 39 min, no significant difference in classification accuracy can be found between the proposed and traditional calibration approach for all of the four modalities.

**Table 2:** Classification results using the traditional and proposed calibration approach.

**Modality**	**Calibration sample size (min)**					
	**13**		**26**		**39**	
	**Proposed**	**Traditional**	**Proposed**	**Traditional**	**Proposed**	**Traditional**
EEG-alone	56.0 ± 10.8	52.0 ± 10.6	61.1 ± 11.1	59.3 ± 11.5	63.4 ± 12.1	62.3 ± 12.5
fNIRS-alone	48.0 ± 10.1	46.1 ± 9.9	51.6 ± 12.0	49.8 ± 12.0	53.9 ± 12.8	51.9 ± 12.8
PHY-alone	41.0 ± 8.0	39.4 ± 8.1	41.8 ± 9.3	40.8 ± 9.5	42.2 ± 10.4	41.8 ± 10.0
EEG+fNIRS	56.9 ± 10.9	53.3 ± 11.0	62.6 ± 11.1	60.8 ± 11.8	65.0 ± 12.3	64.0 ± 12.7
EEG+PHY	55.9 ± 10.8	52.0 ± 10.4	61.1 ± 11.1	59.3 ± 11.4	63.4 ± 12.3	62.2 ± 12.5
fNIRS+PHY	48.7 ± 10.0	46.7 ± 9.7	52.7 ± 11.9	50.6 ± 12.1	55.0 ± 12.6	53.5 ± 13.1
EEG+fNIRS+PHY	56.8 ± 10.9	53.3 ± 10.9	62.5 ± 11.2	60.7 ± 11.7	65.1 ± 12.3	64.1 ± 12.6

*Mean ± standard deviation of the classification accuracies are shown*.

**Table 3:** Effect size and t-statistics of the improvement of the proposed calibration approach over the traditional approach.

**Modality**	**Calibration sample size (min)**	***t*_(20)_**	**Cohen's dz**	**Effect size**
EEG	13	8.66	1.89	Large
EEG	26	4.20	0.92	Large
EEG	39	1.76	0.38	Small
fNIRS	13	5.84	1.27	Large
fNIRS	26	3.63	0.79	Medium
fNIRS	39	2.75	0.60	Medium
PHY	13	3.96	0.87	Large
PHY	26	1.93	0.42	Small
PHY	39	0.77	0.17	Small
EEG+fNIRS	13	6.88	1.50	Large
EEG+fNIRS	26	3.55	0.78	Medium
EEG+fNIRS	39	1.77	0.39	Small

Figure 12 compares the classification accuracy using EEG-alone, fNIRS-alone with those using both EEG and fNIRS. A repeated measures ANOVA revealed that EEG-fNIRS significantly outperforms EEG-alone or fNIRS-alone for both traditional calibration approach and the proposed calibration approach (*p* < 0.001). For the traditional calibration approach, an effect size dz of 0.81 [*t*_(20)_ = 3.70], 0.84 [*t*_(20)_ = 3.83], and 0.85 [*t*_(20)_ = 3.88] has been achieved for a calibration sample size of 13, 26, and 39 min, respectively, when comparing the EEG-alone and EEG+fNIRS approach. For the proposed calibration approach, an effect size dz of 0.89 [*t*_(20)_ = 4.07], 1.18 [*t*_(20)_ = 5.43], and 0.94 [*t*_(20)_ = 4.33] has been achieved for a calibration sample size of 13, 26, and 39 min, respectively, when comparing the EEG-alone and EEG+fNIRS approach. *Post-hoc* analysis was performed using aWilcoxon Signed Rank test with FDR correction comparing EEG-alone and EEG+fNIRS with the results reported in Supplementary Table 5. For all three calibration sample sizes and for both traditional and proposed calibration approaches, EEG+fNIRS significantly outperformed EEG-alone (FDR *q* < 0.05).

The effect of including a physiological-based classifier and combining them with EEG-alone, fNIRS-alone, and EEG-fNIRS classifier was studied and no significant improvement in classification was found.

## Discussion

In this study, the integration of EEG, fNIRS, and physiological measures investigated the classification of three workload levels. To our knowledge, this is the first study that investigated the integration of fNIRS, EEG, and physiological signals for mental workload assessment. The n-back working memory task was adopted to induce three workload levels and the behavioral results suggested successful manipulation of the workload levels.

We first showed that in our data the EEG delta, alpha, low beta, and high beta activities decreased with increased workload levels whereas theta activity increased with an increased workload level at the frontal site Fz. The suppression of alpha power in the posterior areas and increased theta power in the midline frontal areas under workload matches with the results reported in the literature (Gevins et al., 1997). It has been reported that beta activity decreased as workload increased at the midline central site Cz (Gevins et al., 1998). A previous study also suggested that the delta band decreased with increased workload level and the delta band carried information needed to characterize mental workload levels (Zarjam et al., 2011). Our results match those reported in the literature. A concern is that the workload effect on beta activities found in our study maybe caused by motor responses. The effect of workload and key-press type (middle/index finger) was assessed based on the number of key-press within each block and no significant effect of workload and the interaction between workload and key-press type was found. It is possible that motor activities other than key-presses could be affected by workload levels (e.g., subject may be more restless in the low workload condition) which need to be investigated in future studies.

For the fNIRS data, three prefrontal sites were found to be sensitive to workload changes with the 3-back task showing the highest level of activations. Previous fNIRS-based mental workload studies suggested that fNIRS was sensitive to workload changes (Ayaz et al., 2012; Fishburn et al., 2014; Herff et al., 2014). Again, our findings are consistent with the reported results in the literature.

For the physiological data, breath amplitude, breath rate, heart rate, HRV mid band (0.07–0.14 Hz), and HRV high band (0.15–0.5 Hz) were found to be sensitive to workload changes. The suppression of HRV spectral power in the 0.07–0.14 Hz range and 0.15–0.5 Hz range under workload was reported by the literature (Veltman and Gaillard, 1996; Nickel and Nachreiner, 2003). They suggested increased blood pressure and increased heart rate under high workload. Also reported was that breath rate increased with increased workload (Wilson and Eggemeier, 1991). Our results reflected these phenomena.

For workload classification, a significantly better than chance level classification was achieved by all investigated modalities: EEG-alone, fNIRS-alone, physiological alone, and EEG+fNIRS hybrid classification. For improving the classification accuracy when the calibration sample size is small, we proposed to calibrate classifiers using data from both the target subject and a pool of other subjects. Our results indicate that the proposed calibration approach significantly outperformed the traditional calibration approach which only used data from the target subject to calibrate classifiers regardless of the modality adopted. To our knowledge, this was the first study which demonstrated that learning from the data of multiple subjects outperforms learning from a single subject for mental workload decoding accuracy. In the literature, various multisubject learning approaches have been proposed for the classification of different types of tasks. For example, Lotte et al. investigated multisubject learning for the classification of motor imagery tasks (Lotte and Guan, 2010) using EEG. To account for the inter-subject variability, they adopted a data-driven approach to select for each target subject a relevant subset of other subjects whose data can be used to improve the classification of the target subject. Reichert et al. investigated the classification of the phenomenal content of perception using fMRI (Reichert et al., 2014). To achieve cross-subject generalization, the weights of the classifiers trained from individual subjects were combined according to the individual classifier performance. Samek et al. found that the changes between training and testing data is similar across subjects and transferring this non-stationary information between subjects can help improve classification (Samek et al., 2013). Our approach differs from these approaches in that the features for each subject were standardized before training to minimize the inter-subject variability. A further improvement to our approach may be achieved by performing subject subset selection as adopted by Lotte et al. or by weighting the mean and covariance matrices of each subject by their classification performance before applying Equation (3) and Equation (4). Finally, in this study, the hyperparameter λ in Equation (3) and Equation (4) is empirically chosen to be 0.5. The effect of λ on classification accuracies is provided in the Supplementary Figure 1. Estimating λ based on the individual classifier performance and the number of available samples from the target subject may further improve the performance of proposed approach.

Our results also suggest that EEG+fNIRS hybrid classification significantly outperformed EEG-alone or fNIRS-alone workload classification. These findings are consistent with our recent study (Liu et al., 2017) and indicate that there is complementary information about workload in EEG and fNIRS. However, the improvement of EEG+fNIRS over EEG-alone is only about 1–2% in classification accuracy. One possible reason behind this is the relatively low fNIRS-alone performance. It can be seen from Table 2 that fNIRS-alone classification accuracy is about 10% lower than EEG-alone classification. A recent fNIRS-based workload estimation study reports that using only the forehead optodes resulted in a much-reduced workload estimation accuracy compared to using optodes from the whole head (Unni et al., 2017). We speculated that by using whole head coverage, the fNIRS-alone and EEG+fNIRS performance can be much improved. Finally, integrating physiological measures with EEG or fNIRS does not significantly improve workload classification. A reason for the lack of improvement in classification may be due the reduced reliability of the physiological based workload classification in comparison to the brain signal based approaches. Another possibility may be that the physiological measurements do not provide additional information about workload to the brain signal measurements.

In conclusion, the current study presented various approaches for mental workload classification and demonstrated that with the integration of EEG and fNIRS and learning classifiers using the data from other subjects, workload classification performance can be improved. The proposed approaches may have applications in neuroegonomics research and applications such as adaptive aiding systems that are designed to improve the efficiency and safety of human-machine systems during critical tasks.

## Ethics statement

This study was carried out in accordance with the recommendations of the Institutional Review Board of Drexel University with written informed consent from all subjects. All subjects gave written informed consent in accordance with the Declaration of Helsinki. The protocol was approved by the Institutional Review Board of Drexel University.

## Author contributions

YL had the largest contribution for all aspects of the work including the design and programming of the experimental testing, data acquisition, data processing, analysis, and interpretation as well as drafting and editing the manuscript. HA contributed to all aspects of the work with particular emphasis on the fNIRS and EEG application, signal processing, data analysis and interpretation, drafting and editing of the manuscript. PAS contributed to all aspects of the work with particular emphasis with the experimental design, data analysis and interpretation, drafting and editing the manuscript as well as data acquisition. All authors agreed on the content and presentation of the submitted version of the manuscript.

### Conflict of interest statement

The optical brain imaging instrumentation utilized in the present research was manufactured by fNIR Devices, LLC. HA was involved in the development of the technology and thus offered a minor share in fNIR Devices, LLC. The other authors declare that the research was conducted in the absence of any commercial or financial relationships that could be construed as a potential conflict of interest.
