# Supplementary material for: Multisubject “Learning” for Mental Workload Classification Using Concurrent EEG, fNIRS, and Physiological Measures
Source: Front Hum Neurosci. 2017 Jul 27;11:389. doi: 10.3389/fnhum.2017.00389 (PMC5529418; doi:10.3389/fnhum.2017.00389)
Supplement: Supplementary file 1 [file DataSheet1.docx]

Supplementary Material

*of*

Multisubject ‘learning’ for mental workload classification using concurrent EEG, fNIRS and physiological measures

Yichuan Liu^1,2^, Hasan Ayaz^1,2,3,4, *^, Patricia A. Shewokis^1,2,5^

^1^School of Biomedical Engineering, Science & Health Systems, Drexel University, Philadelphia, PA, USA

^2^Cognitive Neuroengineering and Quantitative Experimental Research (CONQUER) Collaborative, Drexel University, Philadelphia, PA USA

^3^Department of Family and Community Health, University of Pennsylvania, Philadelphia, PA USA

^4^The Division of General Pediatrics, Children’s Hospital of Philadelphia, Philadelphia, PA USA

^5^Nutrition Sciences Department, College of Nursing and Health Professions, Philadelphia, PA Drexel University, Philadelphia, PA USA

*** Correspondence:**Hasan Ayaz ([hasan.ayaz@drexel.edu](mailto:hasan.ayaz@drexel.edu))

# Supplementary Figures and Tables

## Supplementary Figures

**
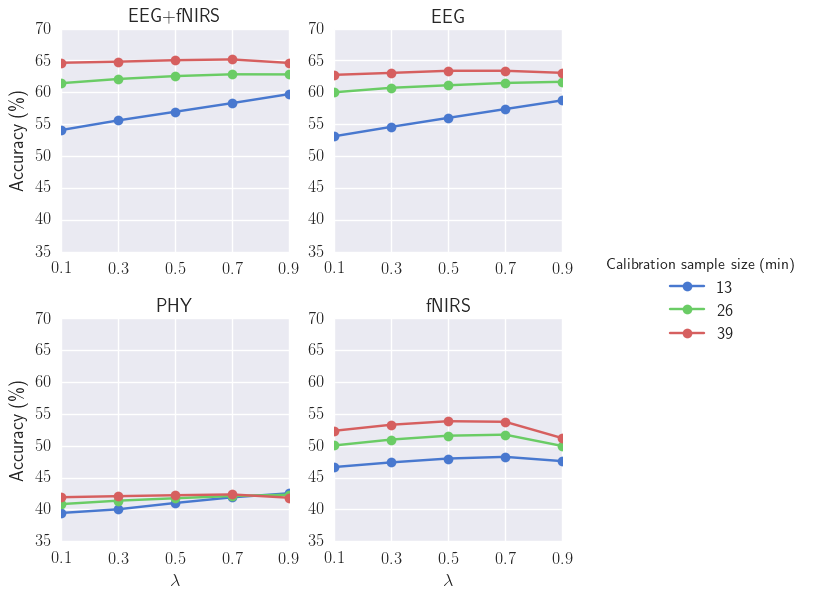
**

**Supplementary Figure 1.** The effect of parameter λ in Equation (3) and Equation (4) of main text on classification accuracies.

## Supplementary Tables

Supplementary Table 1 Means (M) and standard deviations (SD) of the number of key-presses within each block across the 21 participants.

|  | | Key-press type | |
| --- | --- | --- | --- |
|  |  | Left  (M+SD) | Right  (M+SD) |
| nback | 0-back | 11.2±0.3 | 3.8±0.3 |
|  | 2-back | 11.0±0.5 | 3.9±0.4 |
|  | 3-back | 11.2±0.5 | 3.8±0.5 |

Supplementary Table 2 Confusion matrix of classification results adopting the traditional calibration approach using the 39min calibration data. As an example, the first row of the EEG-alone confusion matrix shows that when the true label equals 0-back (i.e. The experimental condition is 0-back), 83.0%, 11.6% and 5.4% of the blocks were predicted by the classification model to be 0-back, 2-back and 3-back, respectively.

| EEG-alone | | Predicted label | | |
| --- | --- | --- | --- | --- |
|  |  | 0-back | 2-back | 3-back |
| True label | 0-back | 83.0 | 11.6 | 5.4 |
|  | 2-back | 6.1 | 52.2 | 41.7 |
|  | 3-back | 2.6 | 46.5 | 50.8 |
| fNIRS-alone | | Predicted label | | |
|  |  | 0-back | 2-back | 3-back |
| True label | 0-back | 54.4 | 29.2 | 16.4 |
|  | 2-back | 24.6 | 51.6 | 23.8 |
|  | 3-back | 16.4 | 33.9 | 49.7 |
| EEG + fNIRS | | Predicted label | | |
|  |  | 0-back | 2-back | 3-back |
| True label | 0-back | 83.2 | 11.7 | 5.1 |
|  | 2-back | 6.1 | 55.6 | 38.2 |
|  | 3-back | 2.5 | 45.2 | 52.3 |

Supplementary Table 3 Confusion matrix of classification results adopting the proposed calibration approach using the 39min calibration data. As an example, the first row of the EEG-alone confusion matrix shows that when the true label equals 0-back (i.e. The experimental condition is 0-back), 86.0%, 9.6% and 4.3% of the blocks has been predicted by the classification model to be 0-back, 2-back and 3-back, respectively.

| EEG-alone | | Predicted label | | |
| --- | --- | --- | --- | --- |
|  |  | 0-back | 2-back | 3-back |
| True label | 0-back | 86.0 | 9.6 | 4.3 |
|  | 2-back | 8.1 | 50.0 | 42.0 |
|  | 3-back | 2.8 | 44.1 | 53.1 |
| fNIRS-alone | | Predicted label | | |
|  |  | 0-back | 2-back | 3-back |
| True label | 0-back | 56.8 | 26.9 | 16.3 |
|  | 2-back | 24.8 | 49.4 | 25.7 |
|  | 3-back | 14.8 | 29.7 | 55.6 |
| EEG+fNIRS | | Predicted label | | |
|  |  | 0-back | 2-back | 3-back |
| True label | 0-back | 86.7 | 9.3 | 4.0 |
|  | 2-back | 8.2 | 52.5 | 39.2 |
|  | 3-back | 2.8 | 42.3 | 54.9 |

Supplementary Table 4. False Detection Rate (FDR)-q value (Benjamini and Hochberg, 1995) testing the difference of classification accuracy between traditional and proposed calibration approaches by adopting a Wilcoxon Signed Rank Test.

|  | Calibration sample size (min) | | |
| --- | --- | --- | --- |
|  | 13 | 26 | 39 |
| EEG | 0.0007 | 0.0117 | 0.1840 |
| EEG+fNIRS | 0.0010 | 0.0256 | 0.1840 |
| PHY | 0.0103 | 0.1840 | 0.4444 |
| fNIRS | 0.0011 | 0.0339 | 0.1293 |

Supplementary Table 5. False Detection Rate (FDR)-q value (Benjamini and Hochberg, 1995) testing the difference of classification accuracy between EEG-alone and EEG+fNIRS approaches by adopting a Wilcoxon Signed Rank Test.

|  | Calibration sample size (min) | | |
| --- | --- | --- | --- |
|  | 13 | 26 | 39 |
| Traditional calibration | 0.0013 | 0.0009 | 0.0018 |
| Proposed calibration | 0.0013 | 0.0006 | 0.0006 |

**Reference**

Benjamini, Y., & Hochberg, Y. (1995). Controlling the false discovery rate: a practical and powerful approach to multiple testing. *Journal of the Royal Statistical Society. Series B (Methodological), 57*(1), 289-300.
